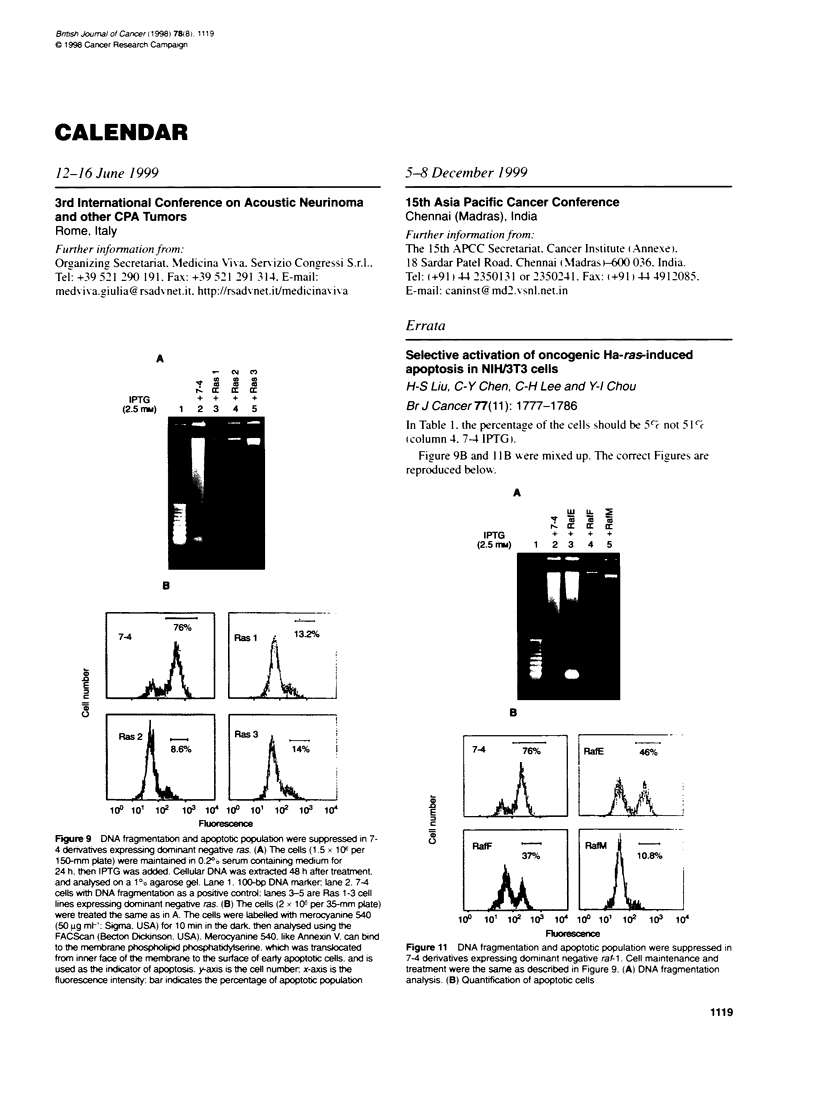# Calendar

**Published:** 1998-10

**Authors:** 

## Abstract

**Images:**


British Journal of Cancer (1998) 78(8), 1119
© 1998 Cancer Research Campaign

### CALENDAR

### *12–16 June 1999*

**3rd International Conference on Acoustic Neurinoma and other CPA Tumors**
Rome, Italy

*Further information from:*
Organizing Secretariat, Medicina Viva, Servizio Congressi S.r.l., Tel: +39 521 290 191, Fax: +39 521 291 314, E-mail: medviva.giulia@rsadvnet.it, http://rsadvnet.it/medicinaviva

### *5–8 December 1999*

**15th Asia Pacific Cancer Conference**
Chennai (Madras), India

*Further information from:*
The 15th APCC Secretariat, Cancer Institute (Annexe), 18 Sardar Patel Road, Chennai (Madras)–600 036, India. Tel: (+91) 44 2350131 or 2350241, Fax: (+91) 44 4912085, E-mail: caninst@md2.vsnl.net.in